# Body mass index, weight change, and risk of second primary breast cancer in the WECARE study: influence of estrogen receptor status of the first breast cancer

**DOI:** 10.1002/cam4.890

**Published:** 2016-10-03

**Authors:** Jennifer D. Brooks, Esther M. John, Lene Mellemkjær, Charles F. Lynch, Julia A. Knight, Kathleen E. Malone, Anne S. Reiner, Leslie Bernstein, Xiaolin Liang, Roy E. Shore, Marilyn Stovall, WECARE Study Collaborative Group, Jonine L. Bernstein, Marinela Capanu, Irene Orlow, Mark Robson, Meghan Woods, John D. Boice, Jennifer Brooks, Patrick Concannon, Dave V. Conti, David Duggan, Joanne W. Elena, Robert W. Haile, Jørgen H. Olsen, Daniela Seminara, Daniel O. Stram, Marc Tischkowitz, Duncan C. Thomas

**Affiliations:** ^1^ University of TorontoDalla Lana School of Public Health SciencesTorontoCanada; ^2^ Cancer Prevention Institute of CaliforniaFremontCalifornia; ^3^ Department of Health Research and Policy (Epidemiology)Stanford University School of MedicineStanfordCalifornia; ^4^ Danish Cancer Society Research CenterCopenhagenDenmark; ^5^ University of IowaIowa CityIowa; ^6^ Lunenfeld‐Tanenbaum Research InstituteMount Sinai HospitalTorontoCanada; ^7^ Fred Hutchinson Cancer Research CenterSeattleWashington; ^8^ Memorial Sloan Kettering Cancer CenterNew YorkNew York; ^9^ Beckman Research InstituteCity of Hope National Medical CenterDuarteCalifornia; ^10^ Department of Population HealthNew York University School of MedicineNew YorkNew York; ^11^ The University of Texas MD Anderson Cancer CenterHoustonTexas

**Keywords:** Body mass index, contralateral breast cancer, estrogen receptor

## Abstract

Studies examining the relationship between body mass index (BMI) and risk of contralateral breast cancer (CBC) have reported mixed findings. We previously showed that obese postmenopausal women with estrogen receptor (ER)‐negative breast cancer have a fivefold higher risk of CBC compared with normal weight women. In the current analysis, we reexamined this relationship in the expanded Women's Environmental Cancer and Radiation Epidemiology (WECARE) Study, focusing on the impact of menopausal status and ER status of the first breast cancer. The WECARE Study is a population‐based case–control study of young women with CBC (cases, *N* = 1386) and with unilateral breast cancer (controls, *N* = 2045). Rate ratios (RR) and 95% confidence intervals (CI) were calculated to assess the relationship between BMI and risk of CBC stratified by menopausal and ER status. Positive associations with obesity and weight gain were limited to women who became postmenopausal following their first primary breast cancer. Among those with an ER‐negative first breast cancer, obesity (vs. normal weight) at first diagnosis was associated with an increased risk of CBC (RR = 1.9, 95% CI: 1.02, 3.4). Also, weight gain of ≥10 kg after first diagnosis was associated with an almost twofold increased risk of CBC (RR = 1.9, 95% CI: 0.99, 3.8). These results suggest that women with an ER‐negative first primary cancer who are obese at first primary diagnosis or who experience a large weight gain afterward may benefit from heightened surveillance. Future studies are needed to address the impact of weight loss interventions on risk of CBC.

## Introduction

Body mass index (BMI) is positively associated with risk of first primary breast cancer in postmenopausal women 1, especially among those who have no history of menopausal hormone therapy (MHT) use 2, 3, 4. Conversely, most studies have found BMI to be inversely associated with premenopausal breast cancer risk 1. The influence of adiposity on hormonal exposures is thought to be the underlying mechanism for these associations 5, 6, 7, although other mechanisms including obesity‐associated inflammation have also been implicated 8.

Approximately 5–10% of breast cancer survivors will go on to develop a second primary tumor in the contralateral breast 9. Risk factors for contralateral breast cancer (CBC) include carrying a *BRCA1* or *BRCA2* mutation 10, 11, 12, 13, having a family history of breast cancer 14, 15, 16, 17, 18, being diagnosed with an estrogen receptor (ER)‐negative first primary breast cancer 19, 20 or one with lobular histology 21, 22, and reproductive factors 23. Breast cancer treatments including chemotherapy 15, 21, 24, 25, 26, 27 and tamoxifen 24, 28, 29, 30, 31 significantly reduce the risk of CBC. Prior studies investigating the association between BMI at first diagnosis and risk of developing CBC have produced mixed results 32, 33, 34, 35, 36, 37, 38. In a recent meta‐analysis, BMI at first diagnosis was positively associated with CBC risk (relative risk = 1.4, 95% CI: 1.2, 1.6, for obese vs. normal weight women) 39. Few studies have examined the impact of menopausal status and ER status on this relationship, or the impact of adult weight change on CBC risk.

The Women's Environmental Cancer and Radiation Epidemiology (WECARE) Study is a population‐based case–control study of young women with CBC (cases) and women with unilateral breast cancer (UBC; controls). Patient recruitment for the WECARE Study took place in two phases (termed WECARE I and WECARE II). In WECARE I, we previously reported that BMI at first diagnosis was not associated with risk of CBC, except among obese postmenopausal women with a first primary ER‐negative breast cancer whose CBC risk was fivefold greater than that of normal weight women 38. The objective of the current analysis was to verify these initial findings and further examine the relationship between BMI and weight change on the risk of CBC in the combined WECARE I and II study population, which effectively doubled our sample size. In this study, particular focus was placed on the impact of menopausal status and ER status of the first primary breast cancer on the relationship between BMI and CBC risk.

## Materials and Methods

### Study population

The WECARE Study is a multicenter, population‐based case–control study of young women where cases are women with asynchronous CBC and controls are women with UBC. Participants were identified through eight population‐based cancer registries: Los Angeles County Cancer Surveillance Program; Cancer Surveillance System of the Fred Hutchinson Cancer Research Center (Seattle, WA); State Health Registry of Iowa; The Cancer Surveillance Program of Orange County/San Diego‐Imperial Organization for Cancer Control (Orange County/San Diego, CA); the Greater Bay Area Cancer Registry (San Francisco Bay Area Region and Santa Clara Region, CA); and the Sacramento and Sierra Center Registry (Sacramento Region, CA). All these cancer registries contribute to the National Cancer Institute (NCI) Surveillance, Epidemiology and End Results (SEER) program in the United States. Patients were also recruited from two other sources, the Ontario Cancer Registry and the Danish Breast Cancer Cooperative Group Registry, supplemented by data from the Danish Cancer Registry. The institutional review board at each recruitment site and the ethical committee system in Denmark and Ontario approved the study. Details of eligibility, recruitment, and study questionnaire have been described previously for WECARE I 40, and were nearly identical for WECARE II.

Briefly, WECARE Study participants were diagnosed prior to age 55 years, between 1985 and 2008 with a first primary invasive breast cancer (stage I–III). Cases were also diagnosed with a second primary CBC (in situ or invasive) at least 1 year later and controls had no history of any other second cancer diagnosis up to their reference date (the date of their matched case's second breast cancer diagnosis). Cases must also have been living in the same study reporting area for both diagnoses, while controls were required to be living in the same cancer reporting area on their reference date as they did for their first breast cancer diagnosis. Additionally, controls must not have undergone prophylactic mastectomy of the contralateral breast. All women had to be alive at the time of contact, able to provide informed consent, complete a telephone interview, and donate a blood or saliva sample for DNA extraction. Controls were matched to cases (1:2 for WECARE I and 1:1 for WECARE II) on year of birth (in 5‐year strata), year of diagnosis (in 4‐year strata), cancer registry region, and race/ethnicity. In WECARE I, cases and controls were further counter‐matched based on the cancer registry reported radiation treatment such that two members of each case–control trio had received radiation treatment for their first breast cancer. Counter‐matching was not used in WECARE II; this was taken into account in all statistical analyses (see below).

Across the eight cancer registries, a total of 2354 CBC cases (998 from WECARE I and 1356 from WECARE II) and 3599 UBC controls (2122 from WECARE I and 1487 from WECARE II) were identified as being eligible and were approached for the study. Of these women, 1521 (65%) CBC cases (708 from WECARE I and 813 from WECARE II) and 2212 (61%) UBC controls (1399 from WECARE I and 813 from WECARE II) completed the phone interview and provided a DNA sample. Reasons for nonparticipation of eligible women included physician refusal (14 cases and 36 controls), interview refusal (768 cases and 1350 controls), and biospecimen refusal (32 cases and 47 controls).

### Data collection

WECARE Study participants were interviewed by telephone using a structured questionnaire which was designed to obtain information about events occurring before the diagnosis of the first primary breast cancer, as well as events that occurred during the at‐risk period (beginning at least 1 year after diagnosis with a first breast cancer and ending at the second diagnosis in CBC cases, or the corresponding date (reference date) for UBC controls). During the structured interview, participants were asked about personal demographics, medical history, family and reproductive history, hormone use, body size, smoking status, and alcohol intake. Additionally, medical records, pathology reports, and hospital charts were used to collect detailed treatment information (i.e., chemotherapy, hormonal therapy, and radiation therapy) for the first primary breast cancer and any recurrences experienced prior to reference date, and tumor characteristics of the first primary tumor (e.g., estrogen receptor (ER) status, histology).

Body size measures were based on self‐report and supplemented as needed with medical record data. These included height and weight at age 18 years, weight at first breast cancer diagnosis, and weight at second diagnosis/reference date. Data at first diagnosis were available from both sources (interview and medical record) for 72% of cases and 78% of controls. Results did not differ when one source was taken first over the other (data not shown). Fifteen women (six cases and nine controls) with missing information from both sources for height and/or weight at first diagnosis, and 22 (nine cases and 13 controls) at reference date were therefore excluded from the analysis. Additionally, women with an implausibly high BMI (>53 kg/m^2^; three controls at first diagnosis and three cases at reference date) or low BMI (<16 kg/m^2^; nine cases and eight controls at first diagnosis, and 15 cases and 11 controls at reference date) were excluded. A further four cases/12 controls and seven cases/11 controls were excluded for having weight values that were outliers (<36.3 kg or >136 kg) at first diagnosis and reference date, respectively (note that there is some overlap between the missing and outlier categories listed above). Women who had reported use of MHT at first diagnosis and/or during the at‐risk period (103 cases and 130 controls) or had unknown menopausal status at first diagnosis or reference date (seven cases and 13 controls) were excluded. After these exclusions, the final analytic dataset included 1386 CBC cases and 2045 UBC controls.

### Statistical analysis

We used conditional logistic regression to generate rate ratios (RR) and 95% CI to assess the relationship between BMI and risk of CBC. Models were adjusted for known risk factors for CBC, including age at diagnosis (continuous), age at menarche (<13 years, ≥13 years, and unknown), number of full‐term pregnancies (nulliparous, 1–3, ≥4, and unknown), first‐degree family history of breast cancer (yes, no, and adopted/unknown), histology (lobular, other, and unknown), stage (local, regional, and unknown), chemotherapy treatment for a first breast cancer (yes, no, and unknown), hormonal treatment for a first breast cancer (yes, no, and unknown), and radiation therapy for a first breast cancer (yes, no, and unknown). A log‐weight covariate was included in the model to account for the sampling probability of the counter‐matching for radiation treatment status used in WECARE I. WECARE II participants (who were not counter‐matched) were assigned an offset term of 1.

Body mass index was categorized as normal weight (<25 kg/m^2^), overweight (from 25 to <30 kg/m^2^), and obese (≥30 kg/m^2^). Weight change between first diagnosis and reference date was calculated and classified as >3 kg loss, 3 kg loss to <3 kg gain, 3 to <10 kg gain, and ≥10 kg gain. Average weight change (kg) per year (during the at‐risk period) was also calculated. Menopausal status at first diagnosis was estimated by comparing the date or age a woman last reported menstruating with the date of her first diagnosis of breast cancer. If a woman reported that she was still menstruating within 2 years before first diagnosis, or was pregnant, she was classified as premenopausal at that time point. A lag of 2 years was used to ensure minimal misclassification caused by rounding errors in the self‐report of age at menopause. Analyses were stratified by menopausal status during the at‐risk period (premenopausal throughout, postmenopausal throughout, and pre‐ to postmenopausal). Women who were classified as pre‐ to postmenopausal are those who were premenopausal at the time of first breast cancer diagnosis, and became postmenopausal during the at‐risk period such that they were postmenopausal at reference date. Analyses were also stratified by the ER status of the first primary breast cancer.

## Results

Table 1 shows selected characteristics of the combined WECARE Study population. Cases and controls are similar with respect to all matching characteristics with a median age of diagnosis of 46 years (range: 23–55 years) and a median age at reference date of 52 years (range: 27–73 years). The majority of women (75% of CBC cases and 76% of UBC controls) were determined to be premenopausal at the time of first diagnosis. Approximately half of all women, 52% of cases and 57% of controls, were diagnosed with an ER‐positive first primary breast cancer.

**Table 1:** Characteristics of CBC cases and UBC controls from the WECARE Study population^a^

Variable	CBC cases*N* = 1386		UBC controls*N* = 2045	
	Median	Range	Median	Range
Age at first diagnosis (years)	46	24–55	46	23–55
Age at reference date (years)	52	27–73	52	27–71
Length of at‐risk period (years)^b^	6.1	1.0–19.8	5.3	1.0–19.8
	*N* (%)	*N* (%)
Study center				
Denmark	255 (18)		431 (21)	
Iowa	186 (13)		294 (14)	
Los Angeles County	189 (14)		356 (17)	
Northern California	297 (21)		316 (15)	
Ontario, Canada	142 (10)		145 (7)	
Orange County	109 (8)		207 (10)	
Seattle	208 (15)		296 (14)	
Year of diagnosis				
1985–1988	221 (16)		434 (21)	
1989–1992	375 (27)		601 (29)	
1993–1996	384 (28)		581 (28)	
1997–2008	406 (29)		429 (21)	
First‐degree family history of breast cancer				
No	912 (66)		1576 (77)	
Yes	456 (33)		433 (21)	
Adopted/Unknown	18 (1)		36 (2)	
Race^c^				
Non‐Hispanic White/Caucasian	1216 (88)		1824 (89)	
Hispanic White	61 (4)		86 (4)	
Black/African American	51 (4)		72 (4)	
Asian	55 (4)		60 (3)	
Other	3 (0)		3 (0)	
Reproductive history				
Menopausal status at first diagnosis				
Premenopausal	1042 (75)		1566 (77)	
Postmenopausal	344 (25)		479 (23)	
Menopausal status during the at‐risk period				
Premenopausal throughout	184 (13)		375 (18)	
Postmenopausal throughout	344 (25)		479 (23)	
Pre‐ to Postmenopausal	858 (62)		1191 (58)	
Menopausal hormone therapy				
Never	1095 (79)		1631 (80)	
Ever (prior to first diagnosis)	284 (20)		408 (20)	
Unknown	7 (1)		6 (0)	
Age at menarche (years)				
<13	655 (47)		882 (43)	
≥13	725 (52)		1155 (56)	
Unknown	6 (0)		8 (0)	
Number of full‐term pregnancies				
None	289 (21)		377 (18)	
1–3	997 (72)		1453 (71)	
4–14	96 (7)		211 (10)	
Unknown	4 (0)		4 (0)	
Characteristics of first breast cancer				
Histology of first breast cancer				
Lobular	161 (12)		205 (10)	
Other	1221 (88)		1837 (90)	
Unknown	4 (0)		3 (0)	
Stage of first breast cancer				
Local	959 (69)		1322 (65)	
Regional	415 (30)		712 (35)	
Unknown	12 (1)		11 (1)	
ER status of first breast cancer^d^				
Positive	729 (53)		1158 (57)	
Negative	424 (31)		516 (25)	
Other	233 (17)		371 (18)	
PR status of first breast cancer^d^				
Positive	633 (46)		999 (49)	
Negative	399 (29)		511 (25)	
Other	354 (26)		535 (26)	
Treatment for first breast cancer^e^				
Received chemotherapy				
No	633 (46)		844 (41)	
Yes	753 (54)		1201 (59)	
Received radiation treatment				
No	591 (43)		484 (24)	
Yes	795 (57)		1560 (76)	
Unknown	0 (0)		1 (0)	
Received hormone treatment^f^				
No	873 (63)		1176 (58)	
Yes	513 (37)		867 (42)	
Unknown	0 (0)		2 (0)	

CBC, contralateral breast cancer; UBC, unilateral breast cancer; ER, estrogen receptor; PR, progesterone receptor.

a Excludes women who were users of menopausal hormone therapy (MHT) at first diagnosis and/or during the at‐risk period (103 CBC cases and 130 UBC controls). Excludes women with missing or implausible body mass index (BMI) or weight at first diagnosis and reference date (date of second diagnosis in cases). Also excludes women with missing menopausal status during the at‐risk period.

b Beginning at least 1 year after first diagnosis extending to the reference date.

c “Asian” includes American Indian, Chinese, Japanese, Filipino, Hawaiian, Korean, Asian Indian, Pakistani, Vietnamese, Polynesian (NOS), Samoan, Other Asian, Pacific Islander (NOS).

d Refers to tumor receptor status of the first primary breast cancer. The “Other” category consists of women where no lab test was given, the test was given, and the results were unknown, or the test was given and the results were borderline.

e Treatments for a first breast cancer and/or recurrence occurring prior to reference date.

f Hormone treatment includes tamoxifen, raloxifene, toremifene citrate, anastrozole, letrozole, exemestane, aminoglutethimide, goserelin acetate, leuprorelin, fulvestrant, megestrol acetate, and fluoxymesterone.

Among women who were premenopausal throughout the at‐risk period, there was a modest inverse association between BMI at first diagnosis and risk of CBC (*P* = 0.04). When compared with normal weight women (BMI < 25 kg/m^2^), this association only reached statistical significance in overweight women (BMI from 25 to < 30 kg/m^2^; RR = 0.5, 95% CI: 0.3, 0.9; Table 2). In women who were premenopausal at first diagnosis and then became postmenopausal during the at‐risk period, no association between BMI at first diagnosis and risk of CBC was observed. However, among women who gained 10 kg or more between first diagnosis and reference date risk was increased relative to women whose weight remained stable during the at risk period (RR = 1.4, 95% CI: 1.01, 1.9; Table 2). When this analysis was further stratified by BMI at first diagnosis (<25, ≥25 kg/m^2^), the association between weight gain and risk of CBC was limited to women with a BMI < 25 kg/m^2^ at first diagnosis (RR = 1.6, 95% CI: 1.1, 2.4; results not shown). Average weight change per year was also modestly associated with risk of CBC, such that a 5% increase in CBC risk was seen per kg gained per year (RR = 1.05, 95% CI: 0.99, 1.10) in women who became postmenopausal during the at‐risk period (results no shown).

**Table 2:** BMI at first diagnosis and weight change between first diagnosis and reference date and risk of CBC, stratified by menopausal status during the at‐risk period

	Premenopausal throughout (*N* = 559)			Postmenopausal throughout (*N* = 823)			Pre‐ to postmenopausal (*N* = 2049)		
	CBC*N* (%)	UBC*N* (%)	RR^a^ (95% CI)	CBC *N* (%)	UBC*N* (%)	RR^a^ (95% CI)	CBC*N* (%)	UBC*N* (%)	RR^a^ (95% CI)
BMI at first diagnosis (kg/m^2^)									
<25	137 (74.5)	259 (69.1)	1.0		305 (63.7)	1.0	598 (69.7)	808 (67.8)	1.0
25 to <30	34 (18.5)	85 (22.7)	0.5 (0.3, 0.9)		121 (25.3)	1.1 (0.8, 1.6)	164 (19.1)	270 (22.7)	0.9 (0.7, 1.1)
≥30	13 (7.1)	31 (8.3)	0.7 (0.3, 1.4)		53 (11.1)	1.0 (0.6, 1.7)	96 (11.2)	113 (9.5)	1.0 (0.7, 1.5)
Weight change^b^ (kg)									
>3 loss	17 (9.2)	38 (10.1)	0.6 (0.3, 1.3)	51 (14.8)	59 (12.3)	1.1 (0.7, 1.9)	90 (10.5)	114 (9.6)	1.1 (0.7, 1.5)
−3 to <3 change	101 (55.5)	195 (52.0)	1.0	150 (43.6)	222 (46.4)	1.0	337 (39.3)	530 (44.5)	1.0
3 to <10 gain	52 (28.3)	104 (27.7)	0.9 (0.5, 1.4)	96 (27.9)	137 (28.6)	1.0 (0.7, 1.5)	296 (34.5)	385 (32.3)	1.2
≥10 gain	14 (7.6)	38 (10.1)	0.5 (0.3, 1.2)	47 (13.7)	61 (12.7)	1.3 (0.8, 2.1)	135 (15.7)	162 (13.6)	1.4 (1.01, 1.9)

CBC, contralateral breast cancer; UBC, unilateral breast cancer; RR, rate ratio; CI, confidence interval; BMI, body mass index (kg/m^2^).

a Adjusted for age at first diagnosis, age at menarche, number of full‐term pregnancies, first‐degree family history of breast cancer, histology, stage, chemotherapy, hormonal therapy, radiation treatment, and ER status of the first primary.

b Weight change between first diagnosis and reference date.

A modest inverse association between BMI at first diagnosis and risk of CBC was observed among women diagnosed with an ER‐positive first primary breast cancer who became postmenopausal during the at‐risk period. The reduced CBC risk associated with obesity was of borderline statistical significance (RR = 0.6, 95% CI: 0.4, 1.01; Table 3). Conversely, risk of CBC was elevated among women diagnosed with an ER‐negative first primary breast cancer who became postmenopausal during the at‐risk period (RR = 1.9, 95% CI: 1.02, 3.4; p for heterogeneity for ER‐positive vs. ER‐negative = 0.01). In this same group of women, those who gained 10 kg or more between first diagnosis and reference date were at increased risk of CBC (RR = 1.9, 95% CI: 0.99, 3.8; p for heterogeneity for ER‐positive vs. ER‐negative = 0.23; Table 3). Again, this association appeared to be confined to women with a BMI < 25 kg/m^2^ at first diagnosis (RR = 3.3, 95% CI: 1.4, 8.1; results not shown). No association between average weight change per year and risk of CBC was seen in analyses stratified by ER status (RR = 1.05. 95% CI: 0.98, 1.12 and RR = 1.06, 95% CI: 0.95, 1.18 in women with ER‐positive and ER‐negative first breast cancers, respectively, p for heterogeneity = 0.88; results not shown).

**Table 3:** BMI at first diagnosis and weight change between first diagnosis and reference data and risk of CBC stratified by menopausal status during the at‐risk period and estrogen receptor (ER) status of the first primary breast cancer

	Premenopausal throughout (*N* = 442)			Postmenopausal throughout (*N* = 688)			Pre‐ to Postmenopausal (*N* = 1697)		
	CBC*N* (%)	UBC*N* (%)	RR^a^ (95% CI)	CBC*N* (%)	UBC*N* (%)	RR^a^ (95% CI)	CBC*N* (%)	UBC*N* (%)	RR^a^ (95% CI)
BMI at first diagnosis (kg/m^2^)									
ER positive									
<25	64 (80.0)	133 (71.1)	1.0	113 (61.1)	176 (61.5)	1.0	320 (69.0)	453 (66.1)	1.0
25 to <30	13 (16.3)	39 (20.9)	0.6 (0.3, 1.2)	51 (27.6)	79 (27.6)	1.1 (0.6, 1.8)	101 (21.8)	160 (23.4)	0.8 (0.6, 1.2)
≥30	3 (3.8)	15 (8.0)	0.3 (0.06, 1.5)	21 (11.4)	31 (10.8)	0.9 (0.5, 1.9)	43 (9.3)	72 (10.5)	0.6 (0.4, 1.01)
ER negative									
<25	41 (63.1)	71 (64.5)	1.0	57 (55.3)	64 (56.1)	1.0	162 (63.3)	194 (66.4)	1.0
25 to <30	16 (24.6)	29 (26.4)	0.6 (0.2, 1.6)	33 (32.0)	32 (28.1)	1.0 (0.5, 2.0)	48 (18.7)	70 (24.0)	0.8 (0.5, 1.4)
≥30	8 (12.3)	10 (9.1)	1.6 (0.5, 5.5)	13 (12.6)	18 (15.8)	0.8 (0.3, 2.3)	46 (18.0)	28 (9.6)	1.9 (1.02, 3.4)
Weight change^b^ (kg)									
ER positive									
>3 kg loss	6 (7.5)	20 (10.7)	0.5 (0.1, 1.4)	29 (15.7)	34 (11.9)	1.4 (0.7, 2.8)	49 (10.6)	75 (10.9)	0.7 (0.4, 1.2)
−3 to <3 kg change	42 (52.5)	101 (54.0)	1.0	83 (44.9)	129 (45.1)	1.0	184 (39.7)	302 (44.1)	1.0
3 to <10 kg gain	26 (32.5)	48 (25.7)	1.0 (0.5, 2.1)	48 (25.9)	81 (28.3)	0.9 (0.5, 1.6)	161 (34.7)	219 (32.0)	1.0 (0.7, 1.5)
≥10 kg gain	6 (7.5)	18 (9.6)	0.5 (0.2, 1.6)	25 (13.5)	42 (14.7)	0.7 (0.4, 1.4)	70 (15.1)	89 (13.0)	1.2 (0.8, 1.9)
ER negative									
>3 kg loss	6 (9.2)	11 (10.0)	0.5 (0.1, 2.6)	18 (17.5)	19 (16.7)	1.2 (0.5, 3.0)	35 (13.7)	27 (9.2)	1.7 (0.8, 3.5)
−3 to <3 kg change	36 (55.4)	51 (46.4)	1.0	41 (39.8)	50 (43.9)	1.0	85 (33.2)	125 (42.8)	1.0
3 to <10 kg gain	15 (23.1)	35 (31.8)	0.6 (0.2, 1.3)	30 (29.1)	33 (28.9)	1.0 (0.4, 2.1)	91 (35.5)	103 (35.3)	1.4 (0.9, 2.3)
≥10 kg gain	8 (12.3)	13 (11.8)	0.4 (0.1, 1.4)	14 (13.6)	12 (10.5)	2.2 (0.7, 6.4)	45 (17.6)	37 (12.7)	1.9 (0.99, 3.8)

CBC, contralateral breast cancer; UBC, unilateral breast cancer; RR, rate ratio; CI, confidence interval; ER, estrogen receptor; BMI, body mass index (kg/m^2^).

a Adjusted for age at first diagnosis, age at menarche, number of full‐term pregnancies, first‐degree family history of breast cancer, histology, stage, chemotherapy, hormonal therapy, radiation treatment.

b Weight change between first diagnosis and reference date.

## Discussion

In this study of BMI at first diagnosis and risk of CBC, we observed a suggestive inverse association with CBC risk among premenopausal women; although when compared to normal weight women, this association only reached statistical significance among those who were overweight at the time of first diagnosis. In women who became postmenopausal during the at‐risk period, CBC risk was increased twofold in women with an ER‐negative first primary breast cancer who were obese at first diagnosis or experienced a large weight gain afterward. These findings confirm the results of our prior analysis from WECARE I 24, providing a more stable estimate of CBC risk among women diagnosed with a first ER‐negative breast cancer.

Several studies have examined the relationship between BMI and risk of CBC. Results from these studies have been mixed, with some finding no association between BMI and risk of CBC 15, 21, 32, 41 and others reporting a positive association 33, 34, 35, 36, 42. Few studies, however, have explored the impact of menopausal and ER status on this relationship. In WECARE I, we found a statistically significant fivefold increased risk of CBC among postmenopausal women with an ER‐negative first primary breast cancer 38. Dignam et al. also found an increased risk of CBC in obese postmenopausal women with ER‐negative first primaries (RR = 2.1) 33. In another study, however, Dignam et al. also found an increased risk in obese women with ER‐positive first primaries (RR = 1.6), regardless of menopausal status 34. We found no evidence of increased CBC risk associated with obesity or large weight gain in women with a ER‐positive first primary breast cancer, whereas other studies of women with an ER‐positive first breast cancer 35 or young women (age < 45 years at first breast cancer diagnosis) 36 found an increased risk of CBC among obese women.

Studies of adult weight change and risk of first primary breast cancer have found weight gain to be positively associated with breast cancer risk among postmenopausal women 43, 44, 45, however, weight gain was not associated 45, 46, 47 or inversely 48 associated with CBC among premenopausal women. To our knowledge, this analysis is the first study to date to examine CBC risk in relation to weight gain since first breast cancer diagnosis. We found a threefold increased risk of CBC associated with large weight gain (≥10 kg) during the at‐risk period in women who became postmenopausal during the at‐risk period, had an ER‐negative first primary breast cancer, and were normal weight at first breast cancer diagnosis. Interestingly, large weight gain has also been associated with increased risk of a first triple negative breast cancer in women <44 years 49 and ER/PR negative breast cancer in postmenopausal women 50. The relatively short interval between first diagnosis and CBC (median length of at‐risk period was 6.1 years for cases and 5.3 years for controls) prevented the assessment of the long‐term impact of weight change on CBC risk, as examined by Majed et al 37.

Chemotherapy 15, 21, 24, 25, 26 and tamoxifen 24, 28, 29, 30 treatments for a first primary breast cancer both reduce a woman's risk of later developing CBC. Prior studies have shown that BMI can influence treatment response 51. We recently published results from the WECARE Study examining the association between adjuvant treatments and risk of CBC 52. Although in this study we found no significant heterogeneity in the relationship between tamoxifen or chemotherapy treatments and risk of CBC by BMI 52, we were unable to examine this relationship further in the current analysis, that is, by stratifying by menopausal and ER status, due to small numbers in these subgroups.

To our knowledge, this is the largest case–control study of body size and risk of CBC conducted to date. This study was of sufficient size that we could perform the necessary stratified analyses to examine the relationship between BMI and risk of CBC within important patient subgroups. Further strengths include the population‐based design where both cases and controls were selected from the same cancer registries, and detailed questionnaire and treatment data available from both interview and medical record review. Limitations include the potential for selection bias given the case–control study design and that not all eligible women participated in the study. However, the recruitment of cases and controls from the same source population (i.e., through cancer registries) will mitigate the potential for selection bias somewhat. Further, because cases and controls were both diagnosed with cancer, participation is less likely to differ by BMI status. Additionally, the proportion of eligible cases and controls who participated in the study was similar suggesting that any potential bias would be nondifferential. Other limitations include the predominantly Caucasian study population, limiting generalizability to other racial/ethnic groups, the lack of data on physical activity, and incomplete data on ER status of the second primary breast cancer, which precluded us from taking these variables into account in the analysis. Further, the majority of women maintained a stable weight, or gained weight, between first diagnosis and reference date with a relatively small proportion of women losing weight between these two time points. We were therefore limited in our ability to assess with confidence the impact of weight loss on the risk of CBC following a first breast cancer diagnosis.

Of particular interest for the current analysis was the examination of the impact of ER status of the first primary breast cancer on the relationship between BMI at first diagnosis and weight change and risk of CBC. Having an ER‐negative first primary breast cancer is itself associated with increased risk of CBC 19, 20. The results of our study suggest that this may be heightened among women who are obese, or those who gain ≥10 kg after being normal weight at first diagnosis. The impact of BMI and weight change on risk of CBC among women with an ER‐negative first breast cancer suggests a potential role for nonhormonal influences of BMI on risk of CBC (e.g., adipokines, chronic inflammation) 8. Future studies investigating these mechanisms and addressing the potential impact of weight loss interventions on risk of CBC could help to reduce the risk of CBC in obese women. Further, the results of this study could help inform more targeted surveillance strategies in this subgroup of at‐risk women.

## Conflict of Interest

None delcared.

## The WECARE Study Collaborative Group

### Memorial sloan kettering cancer center (Coordinating Center) investigators and staff

Jonine L. Bernstein Ph.D. (WECARE Study P.I.); Marinela Capanu Ph.D.; Xiaolin Liang M.D.; Irene Orlow Ph.D.; Anne S. Reiner M.P.H.; Mark Robson, M.D.; Meghan Woods M.P.H..

### Collaborative site investigators

Leslie Bernstein Ph.D.; John D. Boice Jr. Sc.D.; Jennifer Brooks Ph.D.; Patrick Concannon Ph.D.; Dave V. Conti Ph.D.; David Duggan Ph.D.; Joanne W. Elena Ph.D., M.P.H.; Robert W. Haile Dr.P.H.; Esther M. John Ph.D.; Julia A. Knight Ph.D.; Charles F. Lynch M.D., Ph.D.; Kathleen E. Malone Ph.D.; Lene Mellemkjær Ph.D.; Jørgen H. Olsen M.D. DMSc.; Daniela Seminara Ph.D. M.P.H.; Roy E. Shore Ph.D., Dr.P.H.; Marilyn Stovall Ph.D.; Daniel O. Stram Ph.D.; Marc Tischkowitz M.D., Ph.D.; Duncan C. Thomas Ph.D.

### Collaborative site staff

Kristina Blackmore M.Sc.; Anh T. Diep; Judy Goldstein; Irene Harris B.S., C.M.D.; Rikke Langballe M.P.H.; Cecilia O'Brien; Susan Smith M.P.H.; Rita Weathers M.S.; Michele West Ph.D.
